# The GreenBladder Study: Early Detection of Bladder Cancer in Greenland Using a Urinary Biomarker

**DOI:** 10.3390/jcm15020761

**Published:** 2026-01-16

**Authors:** Nathalie Demuth Fryd, Nadja Albertsen, Simon Bernth-Andersen, Andreas Ernst, Jørgen Bjerggaard Jensen

**Affiliations:** 1 Department of Urology, Aarhus University Hospital, 8200 Aarhus, Denmark; natfry@rm.dk (N.D.F.); nadja.albertsen@rm.dk (N.A.); andrerns@rm.dk (A.E.); 2 Department of Clinical Medicine, Aarhus University, 8200 Aarhus, Denmark; 3 Greenland Centre for Health Research, Institute of Health and Nature, Ilisimatusarfik—University of Greenland, Nuuk 3900, Greenland; 4 Department of Surgery, Queen Ingrid’s Hospital, Nuuk 3900, Greenland; sbea@peqqik.gl

**Keywords:** bladder cancer, cystoscopy, urinary biomarkers, point-of-care test, decentralized testing, remote healthcare, Greenland, Arctic

## Abstract

**Background:** Bladder cancer (BC) incidence in Greenland is lower than in other Nordic countries, yet mortality is disproportionately high, suggesting delayed detection. Cystoscopy is the diagnostic gold standard to detect BC, but access in Greenland is often limited by geographic and logistical challenges, underscoring the need for more accessible diagnostic tools. **Objectives:** This study evaluated the performance of the urinary biomarker test Xpert^®^ Bladder Cancer Detection (XBCD) among patients referred for cystoscopy within the Greenlandic healthcare system. **Methods:** In this prospective observational study, 198 patients referred for urological evaluation due to hematuria or other urologic symptoms were recruited from five Greenlandic towns. All participants provided a urine sample for XBCD testing prior to cystoscopy, which served as the reference standard. **Results:** Among 194 patients with valid test results, seven BC cases were detected. XBCD identified five true positives and 166 true negatives, yielding a sensitivity of 71.4%, specificity of 88.8%, and a negative predictive value of 98.8%. **Conclusions:** In this low-prevalence setting, XBCD demonstrated potential as a triage tool to reduce the number of procedures and support earlier BC detection, although findings are limited by the small number of cancer cases.

## 1. Introduction

### 1.1. Bladder Cancer

Bladder cancer (BC) is the ninth most common malignancy worldwide and is among the most resource-intensive cancers to manage [1]. It primarily affects older adults, with a median age of 73, and is about four times more frequent in men. Smoking is the most important risk factor, followed by occupational exposure to carcinogens such as aromatic amines and polycyclic aromatic hydrocarbons [2,3].

The vast majority of BC (>90%) cases are urothelial carcinomas, which are classified as either non-muscle-invasive (NMIBC) or muscle-invasive (MIBC) [4]. NMIBC is often managed with non-radical treatment, including transurethral resection and intravesical therapy. However, there is a high risk of recurrence, with 31–78% of patients experiencing tumor recurrence within 5 years, and 1–45% progressing to MIBC, depending on tumor characteristics and treatment [5]. MIBC is associated with significantly poorer prognosis, higher risk of metastasis at diagnosis, and mortality. Treatment includes radical cystectomy or systemic oncological therapy if metastases are detected [6,7].

Detection of BC at early stages is crucial, as it reduces the need for radical interventions and ultimately improves patient survival outcomes [6,7]. The most common presenting symptom of BC is painless macroscopic hematuria. Other symptoms may include lower urinary tract symptoms (LUTSs) such as urgency and dysuria [8]. Although these symptoms are non-specific and can often be attributed to more benign conditions, the presence of macroscopic hematuria should always prompt timely investigation to rule out underlying malignancy. Currently, cystoscopy is the gold standard for detecting BC [8,9].

### 1.2. Bladder Cancer in Greenland

BC incidence rates show substantial geographic variation, with the highest rates generally seen in industrialized countries. While the Nordic countries are among those with the highest incidences globally, Greenland, which is part of the Kingdom of Denmark, stands out with a notably lower age-standardized incidence rate of 9.8 and 3.5 per 100,000 for men and women, respectively, compared to 20.2 and 6.0 per 100,000 across the Nordic countries. Despite the low incidence, the BC mortality in Greenland appears disproportionately high, as mortality rates remain comparable to the other Nordic countries: 4.7 and 1.9 vs. 4.5 and 1.6 per 100,000 for men and women, respectively [10]. This discrepancy could be caused by underdiagnosis and delayed detection of BC, leading to poorer survival outcomes. This reflects a broader trend, as cancer mortality in Greenland is generally higher than in the rest of the Nordic region, which has been attributed to the unique structural and logistical challenges in the Greenlandic healthcare system [11].

### 1.3. The Healthcare System in Greenland

Greenland is the world’s largest island, covering 2 million square kilometers, yet it has a population of just 56,000 people. Nearly one-third reside in the capital, Nuuk, while the remaining population is distributed across small and remote coastal towns and settlements with no connecting roads [12]. These geographic and infrastructural conditions create substantial logistical barriers to healthcare delivery [13].

While primary care is provided locally across Greenland, more specialized medical services, including urology, are centralized at Queen Ingrid’s Hospital (DIH) in Nuuk, which serves as the primary referral center. All patients in Greenland requiring urologic assessment are referred to DIH, where the urologist triages patients and plans the diagnostics. However, the specialist capacity is limited and waiting times are long, and for those living outside Nuuk, access to urologic care often involves long travel by plane, helicopter, or boat. Consequently, a comprehensive diagnostic workup, including cystoscopy, is often completed during the initial visit. However, this approach is costly and may include unnecessary procedures, which further contributes to long waiting times, delaying diagnosis, particularly for patients residing in remote areas [11,14].

Cystoscopy remains the gold standard for BC detection. However, it is both invasive and resource-intensive, and its limited accessibility in settings like Greenland highlights the need for more accessible and less invasive diagnostic approaches. A potential alternative could be the use of urinary biomarkers. Urinary biomarkers have been an important research area for decades, and a wide range of urine-based biomarker tests have been developed with broad potential clinical applications, including screening, diagnostic work-up and surveillance, but with varying levels of clinical validation [15]. Some of these urinary biomarker tests may offer a non-invasive, decentralized diagnostic approach for early detection of BC. Among these is the Xpert^®^ Bladder Cancer Detection (XBCD), a CE-marked, urine-based real-time quantitative PCR (RT-qPCR) test approved for BC detection in patients with hematuria by measuring the expression of five mRNA biomarkers commonly overexpressed in BC [16].

### 1.4. Study Aim

This study aims to evaluate the utility and diagnostic performance of the XBCD test as an alternative to cystoscopy for BC detection in patients with hematuria and other urinary tract symptoms within the Greenlandic healthcare system.

## 2. Materials and Methods

### 2.1. Study Design

**Figure 1:**
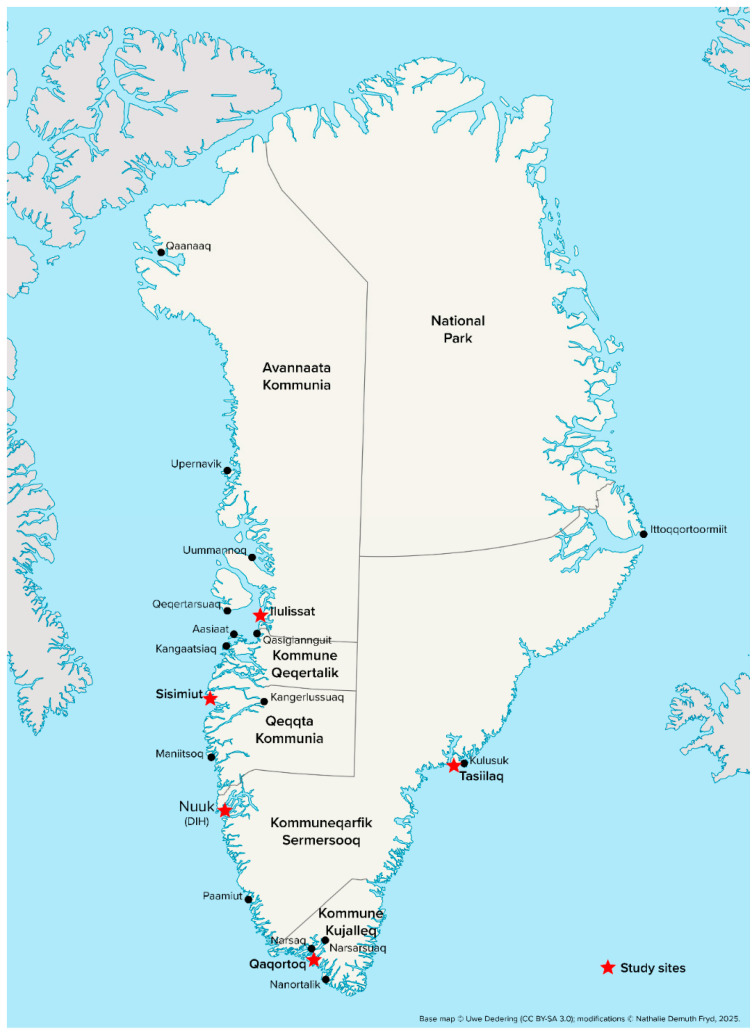
Map of Greenland. Modified from “Greenland edcp location map.svg” by Uwe Dedering (Wikimedia Commons, CC BY-SA 3.0 Unported). Additional color adjustments, city markers, and symbols by the author.

This prospective observational study was conducted between April 2023 and September 2024 across five towns in Greenland: Nuuk, Sisimiut, Ilulissat, Tasiilaq, and Qaqortoq (see Figure 1 for geographic overview). The study was integrated into the existing Greenlandic healthcare framework and carried out in collaboration with the Department of Surgery at DIH in Nuuk and the regional hospitals in Greenland.

### 2.2. Ethics

The study was conducted in accordance with the Declaration of Helsinki and received approval from the Scientific Ethics Committee (reference number: 2023-3605) and the health authorities in Greenland.

All participants provided written informed consent prior to inclusion. Study materials, including participant information, informed consent forms, and questionnaires, were available in both Greenlandic and Danish. In addition, a Greenlandic-Danish interpreter was available during all examinations.

### 2.3. Participants and Recruitment

Participants were recruited from an existing waiting list of patients referred from across Greenland for specialist urologic evaluation during the study period. Eligible participants were adults aged ≥18 years referred for cystoscopy due to hematuria, LUTS, or other urologic symptoms. Importantly, the inclusion of patients without hematuria represents off-label use of the test. Further, inclusion required the ability to understand oral and written information in either Greenlandic or Danish, and to provide written informed consent. Completion of cystoscopy was a criterion for final inclusion. Patients who declined participation or for any reason did not undergo cystoscopy were excluded.

Participants were included in two ways:

(1) During the study period, a mobile study team conducted cystoscopies at temporary satellite clinics established at the local hospitals and healthcare centers in the five selected towns, as part of a collaborative effort to support urologic services and facilitate study recruitment and simultaneously bring down the waiting list. The study team remained in each location for approximately two weeks. Prior to each visit, residents from the respective towns on the waiting list were offered an appointment with the study team at their local hospital. Patients were informed about the study in connection with the invitation and were invited to participate if deemed eligible on the day of the examination. Participation in the study was not a condition for receiving the examination. Patients who for any reason did not attend the appointment remained on the waiting list and were scheduled for evaluation at DIH at a later point in time.

(2) Additionally, patients were included at the urologic outpatient clinic at DIH, where patients from across Greenland on the waiting list were continuously scheduled for specialist urologic evaluation, including cystoscopy, as part of the regular clinical workflow throughout the study period. Eligible patients were invited to participate during pre-consultations for cystoscopy, where a trained nurse handled inclusion and urine sampling. If, upon subsequent evaluation by the urologist, cystoscopy was not performed, patients were excluded from the study.

### 2.4. Study Procedure and Data Collection

Following informed consent, participants completed a brief questionnaire and provided a urine sample for the XBCD test. Cystoscopy was then performed as part of routine diagnostic evaluation and served as the diagnostic reference when compared to the outcome of XBCD.

#### 2.4.1. Baseline Characteristics

Baseline characteristics, including referral information and demographics, were extracted from medical records and patient questionnaire.

Indication for cystoscopy was categorized as either macroscopic hematuria, LUTS, recurrent UTI, or other, based on review of the information provided in the referral letter. If hematuria was mentioned alongside other symptoms, the patient was classified as a hematuria referral.

The questionnaire collected data on self-reported episodes of visible hematuria within the past 12 months and potential BC risk factors, including smoking status and occupational exposure. Occupational exposure was defined as previous employment in any of the following industries: aluminum production, hairdressing, dye manufacturing, printing, painting, textile production, rubber industry, and dry cleaning [17].

All study data were entered and managed in pseudonymized form using REDCap electronic data capture tools hosted at Aarhus University (version 15.5.22) [18,19].

#### 2.4.2. Xpert^®^ Bladder Cancer Detection

The XBCD was performed according to the manufacturer’s protocol (Cepheid, Sunnyvale, CA, USA), as follows: Within 30 min of collection, a urine sample of 4.5 mL is mixed with a stabilization reagent (Xpert^®^ Urine Transport Reagent), allowing storage or transport for up to seven days before testing. After mixing with the stabilization reagent, the urine sample is transferred to a self-contained cartridge (Xpert^®^ Bladder Cancer Detection Cartridge) and inserted into the GeneXpert^®^ system. The analysis runs automatically and requires no further user interaction. After approximately 90 min, the system returns a result of either ‘positive’ or ‘negative’, based on a linear discriminant analysis (LDA) of the cycle threshold (Ct) values from five bladder cancer-associated mRNA targets (ABL1, CRH, IGF2, ANXA10, and UPK1B). LDA is a statistical method that combines the Ct values into a numerical value using a predefined weighting of each target. LDA values exceeding the predefined threshold of ≥0.455 are classified as positive.

If the presence or absence of the target mRNAs cannot be determined, the system reports the result as ‘invalid’. A valid result requires fulfillment of the GeneXpert^®^ system’s built-in quality control, including detection of ABL1 (sample adequacy control), internal control performance, amplification curve quality, and probe check control.

All XBCD analyses were performed on-site in connection with the cystoscopy. A GeneXpert^®^ system was installed at each study site to enable local testing.

For each study subject, the XBCD test result was recorded as either ‘positive’, ‘negative’, or ‘invalid’. For valid test results, the numerical LDA value was also registered.

The XBCD test was conducted for observational purposes only, and its result had no impact on patient management, follow-up, or treatment decisions, which were based solely on local guidelines.

#### 2.4.3. Cystoscopy

Cystoscopy was performed as part of routine clinical evaluation and served as the diagnostic reference standard for this study.

In facilities outside DIH without conventional cystoscope sterilization capabilities, a transportable flexible cystoscope with single-use sheaths or disposable cystoscopes were used.

Cystoscopy findings were categorized as either suggestive or not suggestive of BC. For patients with findings suggestive of malignancy, histopathological confirmation was later obtained by biopsy or TURBT performed at DIH, serving as additional diagnostic verification.

### 2.5. Statistical Analysis

Descriptive statistics were used to summarize baseline patient characteristics either by median, interquartile range (IQR), or mean with standard deviation (SD). The diagnostic performance of the XBCD test was assessed by calculating accuracy, sensitivity, specificity, positive predictive value (PPV), and negative predictive value (NPV), including 95% confidence intervals (95% CI), using cystoscopy findings and histopathologic examination as the reference standard. Subgroup analyses were conducted for (1) patients referred for cystoscopy due to hematuria, and (2) patients with self-reported visible hematuria within the past 12 months.

Analyses were performed as complete-case analyses. Accordingly, patients with invalid XBCD test results were excluded from the diagnostic performance analysis.

All statistical analyses were performed using RStudio (version 2023.06.0+421) [20].

## 3. Results

### 3.1. Study Population

**Figure 2:**
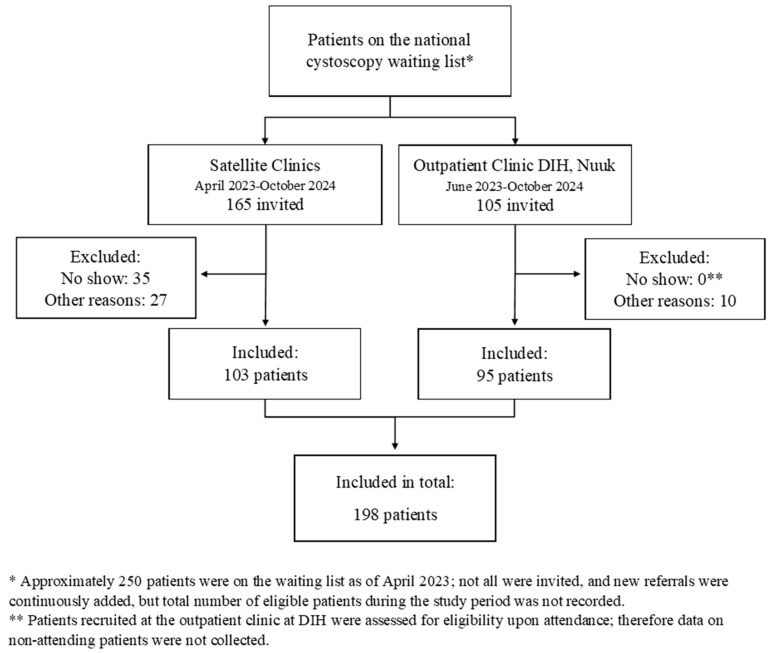
Flow chart inclusion.

In total, 198 patients were included in the study through both recruitment tracks (Figure 2). Additional details on recruitment and geographic distribution of participants are provided in Supplementary Table S1. Between April and September 2023, 165 patients from the five towns were invited to participate in the study through the five satellite clinics (first track). Of these, 35 patients did not attend their scheduled appointment without prior cancelation (‘No Show’). An additional 27 patients did not undergo cystoscopy due to other clinical or logistical reasons (‘Other reasons’) and were therefore not included. Altogether, 103 patients were recruited in this track.

From the second recruitment track, 105 patients from across Greenland were invited via the outpatient clinic at DIH between June 2023 and September 2024. Of these, 10 were excluded as cystoscopy was not performed as initially planned. Baseline demographic and clinical characteristics of the study population are presented in Table 1.

### 3.2. Xpert^®^ Bladder Cancer Detection Test Results

Of the 198 patients included in the study, 194 had a valid XBCD test result and were included in the diagnostic performance analysis (Table 2). Seven had findings suggestive of BC on cystoscopy, of which six were histopathologically verified as urothelial carcinoma. In one case, no biopsy was performed, but cystoscopy findings were consistent with BC and were classified as a true positive case in accordance with the predefined use of cystoscopy as reference standard.

The test correctly identified five true positives and 166 true negatives, with two false negatives and 21 false positives. This corresponds to an overall sensitivity of 71.4% (95% CI: 29.0–96.3), specificity of 88.8% (95% CI: 83.3–92.9), and diagnostic accuracy of 88.1% (95% CI: 82.7–92.3). The positive predictive value (PPV) was 19.2% (95% CI: 6.6–39.4), and the negative predictive value (NPV) was 98.8% (95% CI: 95.8–99.9).

The diagnostic performance of XBCD was also assessed in two subgroups: (1) patients referred for cystoscopy due to visible hematuria (n = 59), and (2) patients who, at the time of cystoscopy, reported visible hematuria within the past 12 months (n = 77). These subgroup analyses are presented in Table 2. Of the seven BC cases, five reported episodes of visible hematuria within the previous 12 months, four of whom had also been referred for hematuria. The remaining two cases had no hematuria mentioned in the referral or by self-report.

Among the five true positive cases, LDA values ranged from 0.4988 to 1.4328, with a median of 0.9875 (IQR: 0.9875–1.0279). Among the two false negatives, one patient had an LDA of 0.4530, just below the predefined cut-off (0.4550), and was found to have an NMIBC low-grade tumor. The other false negative patient had an LDA of 0.1837 and was later diagnosed with disseminated MIBC. False positive results had a median LDA of 0.5617 (IQR: 0.5169–0.5881), while the true negative cases had a median LDA of 0.149 (IQR: 0.0821–0.2492).

## 4. Discussion

### 4.1. Diagnostic Performance of Xpert^®^ Bladder Cancer Detection

In this Greenlandic setting, the XBCD test demonstrated a moderate sensitivity of 71.4% (95% CI: 29.0–96.3) and a high specificity of 88.8% (95% CI: 83.3–92.9). With an NPV of 98.8%, the test shows potential for ruling out BC and thereby sparing some patients from undergoing cystoscopy, whereas the low PPV (19.2%) indicates that a positive result cannot replace cystoscopy for confirming BC in this low-prevalence cohort. Despite the small sample size and the low number of BC cases, the diagnostic performance observed in our study appears broadly consistent with findings from other studies that have evaluated the XBCD in different clinical settings. In the multicenter study by van Valenberg et al. conducted in a hematuria population with a BC prevalence of 7.1%, the test showed a sensitivity of 78% (95% CI: 66–87) and specificity of 84% (95% CI: 81–86) [16]. A prospective multicenter case–control study in Sweden by Abuhasanein et al. reported similar results, with a sensitivity of 79% (95% CI: 73–86) and specificity 83% (95% CI: 76–89) [21]. In a small prospective observational study in patients presenting to the emergency department with macroscopic hematuria, Sordelli et al. reported a sensitivity of 93.8% (95% CI: 69.8–99.8) and a specificity of 51.7% (95% CI: 38.4–64.8) [22]. Although these studies differ in design, patient selection, and disease prevalence, among other factors, making direct comparison difficult, these findings indicate that the test demonstrates relatively stable diagnostic performance across diverse study settings.

Among the seven patients with cystoscopy findings suggestive of BC in our cohort, two were classified as false negatives. One case involved a patient with an NMIBC low-grade tumor and an LDA value of 0.4530, just below the cut-off of 0.4550. This aligns with previous studies reporting lower sensitivity for low-grade tumors, likely due to reduced mRNA biomarker expression and limited tumor cell shedding [21,23]. The other case involved a patient with disseminated MIBC who had an LDA value of only 0.1837, well below the threshold. This is more unexpected as high-grade tumors are generally expected to produce elevated LDA scores and therefore less likely to be missed.

These findings highlight an important limitation of urinary biomarkers for detection: the risk of missed cancers. Abuhasanein et al. addressed this by lowering the LDA cut-off to 0.22 in the Swedish cohort. This increased sensitivity from 79% to 94% but reduced specificity from 83% to 52%, while still allowing 44% of cystoscopies and CT scans to be avoided [21]. However, in Greenland, such an approach is unlikely to provide similar benefits. Given the low prevalence of BC in our cohort and the already notable number of false positives (n = 21), lowering the threshold would mainly add further false positives without substantially improving case detection, thereby limiting the test’s potential to reduce the burden on healthcare resources. This illustrates the trade-off between minimizing the risk of missed cancers and maintaining test specificity to reduce unnecessary procedures. This balance is particularly relevant in settings like Greenland, where access to specialist care is constrained and where logistical costs of unnecessary procedures are high.

### 4.2. Bladder Cancer Risk Factors

A notable characteristic of the study population was the high prevalence of smoking, with nearly 90% of participants being current or former smokers, a well-established risk factor for BC. This indicates a considerable level of exposure to known risk factors within the population, despite the relatively low number of cancer cases detected—and the low incidence of BC in Greenland overall. The study population was also characterized by a relatively young median age compared to the typical BC demographic, where incidence increases markedly with age. The majority of participants were younger than 70 years, and very few were over 80, an age group typically associated with higher BC prevalence. This age distribution may partly reflect the generally low life expectancy in Greenland [11], which could limit the number of individuals living long enough to develop or be diagnosed with BC.

### 4.3. Strengths and Limitations

This study provides unique, real-world data on the diagnostic performance of a POC urinary biomarker in a geographically isolated and sparsely populated setting with distinct healthcare challenges. By prospectively including patients from multiple towns across Greenland and integrating the test within routine clinical workflows, the study captures valuable insights into both test performance and structural barriers in a remote Arctic healthcare system. The inclusion of a mobile cystoscopy team further increased patient inclusion by temporarily expanding cystoscopy capacity beyond routine levels and ensured broader geographic representation.

However, this study is subject to several important limitations. Most notably, the sample size and the number of BC cases were small, which reduced statistical power and the precision of the diagnostic performance estimates. In particular, the predictive values should be interpreted in the context of the low prevalence of BC in our cohort, which inevitably reduces the PPV, while the high NPV largely reflects the already low pre-test probability of BC, limiting conclusions about the test’s added value. While these limitations warrant cautious interpretation, the alignment of our findings with studies from other countries suggests that the test performs similarly in the Greenlandic clinical setting, supporting its broader potential.

Nonetheless, the very low LDA value observed in the case with disseminated MIBC, although based on a single case, also raises the possibility that population-specific biological factors, including genetic differences, could influence biomarker expression and thereby affect diagnostic accuracy. While speculative, it highlights the need for further investigation in larger studies supplemented by studies focusing more specifically on the molecular and genetic differences in susceptibility and tumor characteristics between ethnic groups that might affect the tests applicability.

The XBCD test is primarily validated for use in patients presenting with hematuria, and its diagnostic performance has predominantly been assessed within that clinical context. In this study, however, we applied the test more broadly to all patients referred for cystoscopy for any urologic symptoms. This represents an off-label use of the test and should be acknowledged as such. Our rationale for this approach was driven by both practical and scientific considerations. Restricting the study population to patients with hematuria would have limited inclusion considerably, and an important aim of the study was to explore the test’s performance in a Greenlandic population with distinct genetic and environmental characteristics compared to previously studied populations. While this did not increase the number of BC cases and sensitivity remains limited, it provided a more robust estimate of specificity.

A further limitation relates to the choice of diagnostic reference standard. Cystoscopy was used as the diagnostic reference, reflecting its role as the primary modality for BC detection and its availability in all included patients. To acknowledge the limitations of cystoscopy for definitive diagnosis, histopathological confirmation was obtained in cases with cystoscopic findings suggestive of malignancy and was reported as additional diagnostic verification, where available. As cystoscopy does not provide definitive confirmation nor exclusion of disease in all cases, and given that histopathological verification is only selectively performed, a degree of misclassification due to partial verification bias cannot be fully excluded.

Despite efforts to increase inclusion during the study period, it was not possible to invite all patients on the national cystoscopy waiting list. Remote towns with few eligible patients were not visited due to the logistical and financial burden of traveling with a mobile study setup. As these patients often face greater barriers to both primary evaluation and specialist care [13], this may have introduced a risk of selection bias, although inclusion at the outpatient clinic at DIH likely mitigated this by enabling inclusion of patients from towns not visited by the mobile team (Supplementary Table S1). Still, as continuous monitoring of the waiting list was not feasible, complete data on non-invited patients were not available, leaving some uncertainty regarding potential selection bias. Nevertheless, any overall impact on the findings is expected to be limited, as the proportion of patients not included was small.

Furthermore, four patients were excluded from the diagnostic performance analysis due to invalid test results. An invalid result may arise from various reasons, including technical, pre-analytical, or sample-related factors. Exclusion of these cases may in theory introduce selection bias if the occurrence of invalid results is non-random. However, in the present study, invalid test results were few, and all occurred among cystoscopy-negative patients. As sensitivity is calculated from cases with disease, exclusion of cystoscopy-negative patients cannot influence sensitivity estimates. Any potential impact would therefore be limited to specificity; however, given the small number of invalid results, any such effect would be minimal and would not alter the overall conclusions of the study.

Although not evaluated in the present study, the occurrence of invalid test results is relevant to consider from an implementation perspective, as this may necessitate repeat testing with implications for resource use, logistics, and patient burden.

### 4.4. Clinical Implications and Future Directions

This study reflects the inherent constraints of conducting prospective diagnostic research in geographically isolated and sparsely populated settings such as Greenland. Given the small population and low BC incidence, studies of this type will inevitably be limited by low numbers. Nonetheless, the present study provides valuable real-world data on the applicability of XBCD in a remote Arctic healthcare setting, where structural and logistical barriers to timely diagnosis are substantial. In this context, XBCD may support more effective prioritization of diagnostic evaluations and referrals for further work-up, thereby streamlining the diagnostic pathway for patients with suspected BC. By safely ruling out malignancy in low-risk patients, it may reduce unnecessary cystoscopies. At the same time, any potential use of XBCD for prioritization requires cautious interpretation, ensuring that patients with ongoing clinical suspicion are not subject to unintended diagnostic delay.

Future studies should aim to further explore the test’s role in risk stratification, ideally through multicenter collaborations involving other circumpolar regions with similar cultural and genetic characteristics, such as the Canadian Arctic and Alaska. Such partnerships could support larger-scale evaluations and help determine the broader applicability of biomarker-based tools in Indigenous and remote populations.

## 5. Conclusions

This study evaluated the utility and diagnostic performance of XBCD in Greenland’s unique healthcare setting. Although limited by a small sample size and few BC cases, the test demonstrated a high NPV and accuracy, suggesting its ability to reduce unnecessary procedures by safely ruling out malignancy. However, the possibility of false negatives underscores the continued need for careful clinical judgment and further validation.

In this context, the XBCD test does not offer an alternative for cystoscopy but may offer a practical way to improve diagnostic prioritization and support more equitable access to timely evaluation. Implementing such triage tools should, however, be accompanied by strategies to ensure they are integrated effectively and safely within the existing clinical pathways.

## Figures and Tables

**Table 1:** Baseline characteristics.

	Overall (n = 198)
**Gender, n (%)**	
Male	154 (77.8%)
Female	44 (22.2%)
**Age, years**	
Mean (SD)	58.9 (12.7)
**Place of Residence**	
Nuuk	94 (47.5%)
Outside Nuuk	104 (52.5%)
**Smoking status, n (%)**	
Never Smoked	26 (13.1%)
Former Smoker	80 (40.4%)
Current Smoker	92 (46.5%)
**History of relevant occupational exposure, n (%)**	
Yes	20 (10.1%)
No	178 (89.9%)
**Waiting time (days) from referral to cystoscopy, median (IQR)**	
All referral indications	218 (55–654)
**Referral indication, n (%)**	
Macroscopic hematuria	63 (31.8%)
Lower urinary tract symptoms (LUTS)	100 (50.5%)
Recurrent UTI	24 (12.1%)
Other reason	11 (5.6%)
**Self-reported macroscopic hematuria (past 12 months), n (%)**	
Yes	78 (39.4%)
No	120 (60.6%)

**Table 2:** Diagnostic performance of Xpert^®^ Bladder Cancer Detection against cystoscopy as reference standard.

	All Cases (n = 194)	Hematuria Referrals (n = 59)	Self-Reported Hematuria (n = 77)
**True positives**	5	3	4
**True negatives**	166	45	58
**False positives**	21	10	14
**False negatives**	2	1	1
**Sensitivity (95% CI)**	71.4% (29.0–96.3)	75.0% (19.4–99.4)	80.0% (28.4–99.5)
**Specificity (95% CI)**	88.8% (83.3–92.9)	81.8% (69.1–90.9)	80.6% (69.5–88.9)
**PPV (95% CI)**	19.2% (6.6–39.4)	23.1% (5.0–53.8)	22.22% (6.4–47.6)
**NPV (95% CI)**	98.8% (95.8–99.9)	97.8% (88.5–99.9)	98.3% (90.9–99.9)
**Accuracy (95% CI)**	88.1% (82.7–92.3)	81.4% (69.1–90.3)	80.5% (69.9–88.7)

## Data Availability

The datasets generated in this study are subject to legal and ethical restrictions and cannot be made publicly available. According to the project approval, individual-level data may only be used within the approved study and cannot be shared without prior authorization from the Greenlandic Health Authorities.

## Supplementary Materials

The following supporting information can be downloaded at: https://www.mdpi.com/article/10.3390/jcm15020761/s1, Table S1: Participants by place of residence and recruitment track.

## Abbreviations

The following abbreviations are used in this manuscript:

BC	Bladder cancer
NMIBC	Non-muscle invasive bladder cancer
MIBC	Muscle invasive bladder cancer
LUTS	Lower urinary tract symptoms
DIH	Dronning Ingrids Hospital (Queen Ingrids Hospital)
XBCD	Xpert^®^ Bladder Cancer Detection
RT-qPCR	Real-time quantitative PCR
LDA	Linear discriminant analysis
Ct	Cycle threshold
PPV	Positive predictive value
NPV	Negative predictive value
TURBT	Transurethral resection of bladder tumor
