# Supplementary material for: The GreenBladder Study: Early Detection of Bladder Cancer in Greenland Using a Urinary Biomarker
Source: J Clin Med. 2026 Jan 16;15(2):761. doi: 10.3390/jcm15020761 (PMC12842550; doi:10.3390/jcm15020761)
Supplement: Supplementary file 1 [file jcm-15-00761-s001.zip › jcm-4061043-supplementary.pdf]

Supplementary Materials

Supplementary Table S1. Participants by place of residence and recruitment track

Place of residence	Satellite Clinics (n)	Outpatient Clinic DIH (n)	Total (n)
Nuuk	46	48	94
Tasiilaq	6	0	6
Sisimiut	26	5	31
Ilulissat	14	6	20
Qaqortoq	11	4	15
Aasiaat	0	9	9
Upernavik	0	3	3
Ummannaq	0	4	4
Maniitsoq	0	2	2
Paamiut	0	1	1
Narsaq	0	4	4
Nanortalik	0	2	2
Kangerlussuaq	0	1	1
Qaarsut	0	1	1
Qeqertarsuaq	0	1	1
Qaanaaq	0	3	3
Narsarsuaq	0	1	1
Total	103	95	198
